# Using lessons from criminal justice research to improve conservation law enforcement research and practice

**DOI:** 10.1111/cobi.70094

**Published:** 2025-07-03

**Authors:** Freya A. V. St. John, Leejiah Dorward, Harriet Ibbett, Martina Feilzer

**Affiliations:** ^1^ School of Environmental and Natural Sciences Bangor University Gwynedd UK; ^2^ School of History, Law & Social Sciences Bangor University Gwynedd UK

**Keywords:** deterrence, law enforcement, legitimacy, procedural fairness, protected areas, rangers, wildlife crime, aplicación de la ley, áreas protegidas, crímenes de fauna, disuasión, equidad procesal, guardabosques, legitimidad, 威慑, 执法, 合法性, 程序公正, 保护地, 护林员, 野生动物犯罪

## Abstract

Urgency to save species from extinction has prompted increased investment in law enforcement in protected areas. To date, such law enforcement has largely focused on increasing costs and reducing opportunities for offending. However, these resource‐intensive approaches are not always effective and can contribute to conflict between people and conservation authorities, undermining human well‐being and conservation goals. Drawing on criminal justice research, we considered how procedural justice theory—which examines how fair process and the perceived legitimacy of rules and enforcers influence behavior— could enhance understanding of compliance dynamics and complement existing law enforcement approaches, particularly in addressing low‐level noncompliance in protected areas. We also explored how principles of procedural justice have been incorporated in general policing and outlined challenges and opportunities to integrating this approach into conservation law enforcement. We considered key opportunity‐based (e.g., routine activity theory) and actor‐based frameworks (e.g., deterrence theory) underpinning most protected areaenforcement. We then focused on procedural justice theory and the role of legitimacy in encouraging compliance. Evidence from general policing shows that when enforcers treat citizens fairly, listen, and make decisions objectively, they gain trust and legitimacy. In turn, people are more inclined to comply with laws and cooperate with enforcers. Procedural fairness can be implemented during encounters by embracing 4 pillars: neutrality, voice, respect, and trustworthiness. Outlining challenges of integrating this approach in conservation law enforcement, we highlight the need to address limited public trust in state authority and other factors including working conditions of enforcers. Alongside ensuring the integrity and accountability of conservation law enforcement, we argue that embedding principles of procedural fairness into interactions between enforcers and citizens could reduce low‐level noncompliance. Success, however, requires conservation law enforcement to be reconceptualized by placing procedural fairness and legitimacy on a more equal footing with deterrence in research and practice.

## Abstract (es)

Aprendizajes de la investigación en justicia penal para incrementar la investigación y práctica de la aplicación de las leyes de conservación

**Resumen:**

La urgencia por salvar especies de la extinción ha impulsado una mayor inversión en la aplicación de la ley en las zonas protegidas. Hasta la fecha, esta aplicación de la ley se ha centrado en gran medida en aumentar los costos y reducir las oportunidades de delinquir. Sin embargo, estos enfoques, que consumen muchos recursos, no siempre son eficaces y pueden contribuir al conflicto entre las personas y las autoridades de conservación, disminuyendo el bienestar humano y los objetivos de conservación. Con base en la investigación sobre justicia penal, analizamos cómo la aplicación de la teoría de la justicia procedimental ‐que examina cómo influyen en el comportamiento un proceso justo y la legitimidad percibida de las normas y los responsables de su cumplimiento‐ en la investigación sobre conservación podría mejorar la comprensión de la dinámica del cumplimiento y complementar los enfoques existentes de aplicación de la ley, en particular para abordar el incumplimiento de bajo nivel en las áreas protegidas. También analizamos cómo se han incorporado los principios de la justicia procesal a la actuación policial en general y esbozamos los retos y las oportunidades de integrar este enfoque en la aplicación de la legislación sobre conservación. Examinamos los principales marcos basados en la oportunidad (por ejemplo, la teoría de la actividad rutinaria) y en los actores (por ejemplo, la teoría de la disuasión) que sustentan la mayor parte de la aplicación de la ley en las áreas protegidas; la teoría de la justicia procesal; y el papel de la legitimidad a la hora de fomentar el cumplimiento. Los datos sobre la actuación policial en general demuestran que cuando los encargados de hacer cumplir la ley tratan a los ciudadanos con justicia, escuchan y toman decisiones objetivas, ganan confianza y legitimidad. A su vez, la gente está más dispuesta a cumplir las leyes y a cooperar con las fuerzas del orden. La justicia procesal puede aplicarse durante los encuentros mediante cuatro pilares: neutralidad, voz, respeto y fiabilidad. La inclusión de este enfoque en la aplicación de las leyes de conservación supone un reto, y es necesario abordar la limitada confianza pública en la autoridad estatal y las condiciones de trabajo de los encargados de hacer cumplir la ley. Además de garantizar la integridad y la rendición de cuentas de la aplicación de la legislación en materia de conservación, sostenemos que la incorporación de principios de equidad procesal en las interacciones entre las fuerzas del orden y los ciudadanos podría reducir los incumplimientos de bajo nivel. Sin embargo, para tener éxito, es necesario volver a conceptualizar la aplicación de la legislación en materia de conservación y colocar la equidad y la legitimidad de los procedimientos en pie de igualdad con la disuasión en la investigación y la práctica.

## 利用刑事司法研究的经验教训来改进保护执法研究与实践

**【摘要】** 拯救濒临灭绝物种的紧迫性已促使人们在保护地执法方面加大投入。迄今为止, 此类执法主要聚焦于提高犯罪成本和减少犯罪机会。然而, 这些资源密集型的方法并不总是有效, 且可能加剧人们与保护机构之间的冲突, 从而破坏人类福祉和保护目标。我们借鉴刑事司法研究, 分析了如何将程序正义理论(该理论检验了公平的程序及人们对规则及执法者合法性的认知对行为的影响)应用于保护研究, 以加强对守法行为动态的理解并补充现有的执法方法, 尤其是在处理保护地中低水平违法行为的方面。我们还探讨了程序正义原则在一般执法活动中的应用, 并概述了将此方法纳入保护执法所面临的挑战与机遇。研究分析了支撑大多数保护地执法行为的机会导向(如常规活动理论)和行为者导向(如威慑理论)的重要框架、程序正义理论, 以及合法性在促进守法中的作用。一般执法活动的证据表明, 当执法者公平对待公民、倾听意见并客观决策时, 他们能获得人们信任和对其合法性的认可。相应地, 人们也更倾向于遵守法律并配合执法者。在执法过程中践行程序公正有四大支柱:中立性、表达权、尊重和可信度。将这一方法融入保护执法具有挑战性, 且需解决公众对国家权威信任不足及执法者工作条件等问题。在确保保护执法的完整性和责任性的同时, 我们认为将程序正义原则纳入执法者与公民的互动中, 可减少低水平违法行为。然而, 该方法的成功还有赖于在研究与实践中将程序公正及合法性置于与威慑同等重要的地位, 来重新思考保护执法的概念。**【翻译:胡怡思;审校:聂永刚】**

## INTRODUCTION

The urgency to save species from extinction has prompted an increased focus on law enforcement in conservation. Each year, considerable sums are invested in antipoaching approaches and punitive sentencing policies (Duffy et al., 2019; Wilson & Boratto, 2020). In some contexts, these efforts have become increasingly top‐down and militarized as pressure mounts on states to deter organized wildlife crime (Hübschle, 2017). For example, millions of dollars were recently spent on strengthening efforts to reduce illegal wildlife trade globally, with funds used to arm and train enforcers and support efforts to increase fines and prison sentences (Paudel et al., 2020; World Bank, 2016). Yet, evidence demonstrating the effectiveness of such approaches in conservation is limited (Boratto & Gibbs, 2021; Wellsmith, 2011). More broadly, meta‐analyses from the general crime literature show that tough‐on‐crime policies rarely deter rule breakers (Dölling et al., 2009; Pratt et al., 2006). Moreover, conservation authorities’ focus on crime statistics as key performance indicators (e.g., number of patrols, arrests, confiscations) risks law enforcement agents, such as park guards, rangers, police, or army (hereafter enforcers), focusing disproportionate effort on easy targets, such as those attempting to access natural resources for subsistence purposes (Herbig, 2008; Hübschle et al., 2021).

Noncompliance in conservation ranges from low‐level transgressions associated with subsistence needs (e.g., collection of plant resources for household use; subsistence hunting [McElwee, 2010; Tranquilli et al., 2014]) to organized crime targeting high‐value commodities. In protected areas of highly biodiverse countries, enforcers regularly tackle the former (Kisingo et al., 2022; McElwee, 2010), with those most affected often among the poorest and most marginalized, raising questions about the equity and justice of commonly applied approaches to conservation law enforcement (Crosman et al., 2022). Although a variety of different policing approaches exist more broadly and in conservation (such as community, problem‐oriented [Moreto & Charlton, 2021], and intelligence‐led policing [Moreto et al., 2018]), these often focus on harsh enforcement and sentencing. Yet, these can reproduce existing injustices and confound other power imbalances (Duffy et al., 2016). Steep fines increase social inequalities (Paudel et al., 2020), and imprisonment is costly to individuals, families, and the state and is often unsafe and dysfunctional in many biodiverse countries (Dore et al., 2022; Institute for Crime & Justice Policy Research, 2023). Critically, when laws and societal values conflict, such as when rules criminalize resource use previously governed by social norms (Jones et al., 2008), or restrict access to vital means of subsistence (Roe, 2008), tension can arise between authorities and citizens (Wilson & Boratto, 2020). For example, if people hunt protected wildlife to meet basic needs and conservation authorities treat the activity like hunting illegally for commercial purposes (e.g., ivory trafficking), people may view authorities as uncaring, arbitrary, and untrustworthy (Moreto & Gau, 2017). This can contribute to conflict between people and conservation authorities, reducing compliance and undermining both human well‐being and biodiversity outcomes.

Evidence from criminal justice research suggests that when enforcers treat citizens fairly, listen, and make decision objectively, they gain trust and legitimacy. In turn, people are more inclined to comply with laws, cooperate with, and help enforcers (Moreto & Gau, 2017; Tyler, 2017; Tyler & Jackson, 2013). However, limited attention has been given to the nature of encounters between enforcers and citizens in conservation. We considered how the application of procedural justice theory in conservation research could enhance understanding of compliance dynamics and thus improve and complement existing approaches to conservation law enforcement, particularly, although not exclusively, when addressing low‐level noncompliance in protected area contexts.

In the conservation literature, several reviews synthesize causes of rule breaking and review interdisciplinary literature on compliance (e.g., Moreto & Elligson, 2025; Oyanedel et al., 2020b; Ramcilovic‐Suominen & Epstein, 2012). Typically, such reviews cover theories that can be assigned to one of 2 categories: opportunity‐based approaches that focus on altering the immediate environment to decrease opportunities for offending or actor‐based approaches that focus on addressing motivations of individual actors by considering instrumental, normative, or legitimacy factors. Not wishing to duplicate prior reviews, we briefly outlined key theories underpinning the majority of law enforcement practice in protected area contexts (i.e., opportunity‐based approaches and deterrence theory) (Gore, 2017; Lemieux, 2014) and then focused on the less‐studied role of legitimacy in encouraging conservation compliance. Drawing on examples from criminal justice research, we reflected on how a stronger focus on value‐based approaches to compliance, grounded in the theoretical and analytical framework of procedural justice theory, could further enhance understanding of compliance in conservation. We also explored the challenges and opportunities for integrating procedural justice theory into conservation law enforcement practice.

## COMMON APPROACHES TO LAW ENFORCEMENT CURRENTLY ENACTED IN CONSERVATION

### Opportunity‐based approaches

Grounded in the premise that noncompliance is mostly a product of opportunity for motivated individuals (Brantingham & Brantingham, 1981; Clarke & Felson, 2017; Oyanedel et al., 2020b; Wortley & Townsley, 2017), opportunity‐based theories focus on the targets of crime and emphasize the ecological nature of criminal acts. Such theories are based on arguments that crimes are an interaction between an offender, targets, and the guardians who protect potential targets from crime (i.e., enforcers, but also regular citizens). In response, opportunity‐based theories of enforcement are based on the assumption that noncompliance is distributed nonrandomly across space and time and focus on altering the immediate environment to decrease opportunities for offending, thus increasing compliance (Oyanedel et al., 2020b; Wortley & Townsley, 2017).

**FIGURE 1:**
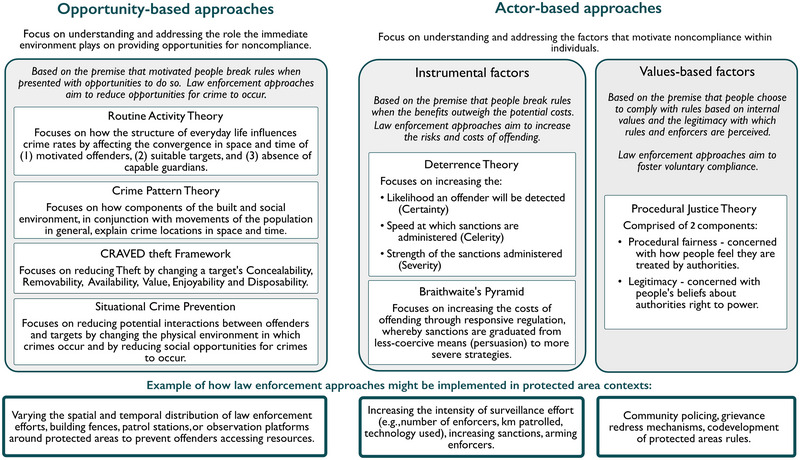
Schematic outlining some of the theories underpinning approaches to law enforcement. Only theories we discuss are included.

In their seminal article, Cohen and Felson (1979) used routine activity theory to explain why urban crime rates in the United States increased in the 1960s despite the fact that conditions presumed to cause crime (e.g., poverty, lack of education, unemployment) had generally improved (Figure 1). To explain this, they focused on determining how changes in peoples’ daily activity patterns provide greater opportunity for crimes against persons or property (Felson & Cohen, 1980; Miro, 2014). They found that transformations in modern society, including women's participation in the labor force, higher college enrolment, and longer vacation lengths, increase opportunities for possible offenders as more homes were left unattended. Technological advancements, which saw an increase in the ownership of desirable electronics (e.g., televisions and stereos) and thus the quantity of suitable targets lacking guardians to prevent crimes, created an environment of increased criminal opportunity (Miro, 2014). Similarly, crime pattern theory considers environmental cues or opportunities for crime rooted in the physical and social features of the environment and suggests that by understanding daily activity patterns opportunistic crimes can be disrupted (Brantingham, 2016; Brantingham & Brantingham, 1993).

Aligning these theories, situational crime prevention combines routine opportunity theory with the rational choice model of offending by considering the physical environments hosting crime and the actors weighing up the costs and benefits of committing a crime (Clarke, 1995; Eck & Clarke, 2019). It considers how places can be altered to reduce offender–target interactions or, if interactions are unavoidable, to increase guardianship of potential targets both formally, by deploying enforcers capable of protecting targets, and informally by drawing on the influence that respected others have on potential rule breakers (Lemieux, 2014). Building on this premise, the CRAVED theft framework assesses theft preferences by considering a target's concealability, removability, availability, value, enjoyability, and disposability. This framework has been used to help explain environmental crimes, including parrot theft in Mexico (Pires & Clarke, 2011) and illegal commercial fishing (Petrossian & Clarke, 2014), and informs situational crime prevention strategies aimed at increasing compliance by reducing criminal opportunity. Lemieux (2014) provides a detailed explanation of criminal opportunity theory and the application of situational crime prevention to illegal wildlife extraction with case studies from South Africa, South America, and Malaysia, among others. Further, Kurland et al. (2017) applied a situational crime prevention lens to their review of wildlife crime literature to determine whether different types of wildlife crimes cluster and to identify whether conservation interventions for reducing crime mirrored those in situational crime prevention.

Reducing opportunities for motivated offenders is certainly a potential route for securing compliance in many conservation contexts. However, success depends on the ability of conservation authorities to alter the immediate environment to decrease opportunities for offending to occur. Yet, doing so, particularly across vast protected area landscapes neighbored by people who may be disenfranchised by conservation or the state more generally, can be physically, socially, and economically challenging. Indeed, numerous studies show that current enforcement strategies are significantly hindered by insufficient investment in enforcer personnel and resources (e.g., Appleton et al., 2022; Gray et al., 2024).

### Actor‐based approaches and deterrence theory

Rooted in a rational choice framework, classic deterrence theory suggests that would‐be offenders weigh up costs and benefits of noncompliance when deciding how to act (Moreto & Gau, 2017; Pratt et al., 2006). This actor‐based theory is composed of 3 elements: the likelihood of capture, sanction severity, and the speed at which sanctions are applied (Figure 1). Accordingly, certain, swift, and severe application of laws should create both specific deterrence, whereby offenders refrain from criminality because of their sanction experience, and general deterrence, whereby awareness of sanctioning encourages compliance among others (Wilson & Boratto, 2020). Such an approach resonates with the belief that people avoid actions that have costly consequences (Pratt et al., 2006). The creation of deterrence hinges on the ability of authorities to detect and punish offenders or at least creates perceptions that offenders will be detected and punished (Stern, 2008).

Despite being widely applied, various factors, including resource constraints (Hilborn et al., 2006), detection challenges (Ibbett et al., 2020), political will (Lundquist & Granek, 2005), and the ingenuity of offenders to evade detection or circumvent conservation rules (Stern, 2008) restrict the potential of enforcers to secure deterrence‐based compliance with conservation rules. Meta‐analyses conducted in criminology to investigate the effectiveness of deterrence theory concluded that variables specified by the theory (e.g., certainty and severity of punishment) are at best weak predictors of compliance (Pratt et al., 2006). Evidence from conservation law enforcement echoes these findings. Analyses of catch per unit effort data collected by protected area rangers suggest that deterrence, if detectable, is often small, local, and short‐lived (Dobson et al., 2019). For example, analysis of enforcement effort in 4 terrestrial parks across tropical Africa and Southeast Asia provides only weak support that ranger patrols discourage illegal activities, and they discourage them only in the short term (Dancer et al., 2022). In recognition of such limitations, writing on general crime, Ayres and Braithwaite (1992) argue that for regulations to be effective, there needs to be synergy between persuasion and punishment. They propose responsive regulation that signals to would‐be offenders the capacity to escalate enforcement (Arias et al., 2015; Ayres & Braithwaite, 1992). Their approach is conceptualized as a pyramid, with less coercive interventions at the base (e.g., persuasion) and more severe strategies higher up. They argue that graduated sanctioning is likely to be deemed more legitimate than applying overtly strong sanctions immediately, which can have adverse effects, including increased chance of bribery and violence (Arias, 2015).

Although securing compliance through deterrence may appear simple, it obliges authorities to produce benefits and exercise coercion every time they seek to influence people's behavior (Tyler, 2006). However, protected areas often span vast areas, making such strategies resource intensive and challenging to implement (Moreto, 2016). As exemplified by the case of the Sulawesi babirusa in Indonesia, where traders gradually became habituated to enforcement efforts (Milner‐Gulland & Clayton, 2002), altering public behavior by threatening sanctions and reducing opportunities to offend is challenging. Over time, a focus on sanctions alone undermines peoples’ relationships with authorities and so requires ever‐increasing resources (Tyler, 2017; Tyler & Jackson, 2013). Additionally, challenges may worsen during periods of resource scarcity or crisis (Tyler, 2017), as illustrated in the Serengeti National Park (Hilborn et al., 2006). At such times, stressed regimes may be less able to maintain law enforcement operations and thus depend more on public support to generate compliance, despite the prospect of detection and punishment being low. However, such deference cannot be assumed, particularly where citizens associate protected area authorities with historic or contemporary injustices, as is often the case in protected area contexts of fragile, transitional states where much conservation occurs.

Recent theoretical revisions to deterrence theory indicate that an individual's cost–benefit analysis is linked to a variety of factors, some of which are related to deterrence (e.g., direct or indirect experience of enforcement), whereas others, such as an individual's ability to exercise self‐control in the face of temptation, are not (Pratt et al., 2006). In contrast to what rational choice conceptualizations of human behavior predict, people obey laws when the likelihood of punishment is virtually zero, yet commit infractions that involve substantial risk of detection and punishment (Tyler, 2006). Moreover, focusing on deterrence theory when addressing illegal hunting can result in policies too focused on sanctions (Nurse, 2011; von Essen et al., 2014) rather than other motivating factors. Explanations of crime based solely on deterrence theory overlook the actions of those who believe they have no choice but to transgress to meet basic needs, due to power differentials, or to protest unjust laws (Moreto & Gau, 2017). Finally, calls for increased enforcement ignore the fact that many people willingly follow the law without being forced to by authorities (Stern, 2008).

### Actor‐based approaches and value‐based compliance

Value‐based approaches acknowledge that compliance is influenced by peoples’ internal values, the strength of which can exceed that of the perceived risk of being caught and sanctioned (Sunshine & Tyler, 2003; Trinkner et al., 2018). Values reflect people's assessments of what is appropriate in a given situation and involves people's feelings of responsibility to others (Tyler, 2017). Value‐based theories of compliance propose that where individual actors believe laws should be obeyed, they comply because it is the right thing to do, not because they fear capture or punishment (Moreto & Gau, 2017). Due to the psychological process of internalization (whereby over time society's moral norms become part of a person's internal motivation system guiding their behavior), value‐based motivations can produce consistent behavior across situations and time (Manfredo, 2008; Tyler, 2017). Thus, where people feel obliged to obey rules, their behavior should reflect this value irrespective of changes in costs and rewards presented across settings (Tyler, 2017).

The inclusion of value‐based factors, such as social influence and morality, has resulted in superior models of compliance behavior in, for example, studies of Malaysian fishers (Kuperan & Sutinen, 1998). Drawing on this work, Viteri and Chavez (2007) tested an econometric model of fisher compliance combining traditional deterrence measures with boat owners’ perceptions of the legitimacy of regulations. Results showed that artisanal fishers of the Galapagos are less inclined to commit infractions when they perceive rules as legitimate and believe leaders of their organization represent their interests (Viteri & Chávez, 2007). Moreover, results of an empirical analysis of fuelwood harvests suggest that involving resource users in rulemaking and enforcement significantly increases compliance (Epstein, 2017). Evidence from 12 marine protected areas in Costa Rica similarly suggests that meaningful involvement in decision‐making encourages compliance alongside other factors, including tourism levels and perceptions of the effectiveness of government efforts against illegal fishing (Arias et al., 2015). However, involvement in decision‐making does not always trigger compliance, as illustrated among fishers in Brazil, Indonesia, and Philippines (McDonald et al., 2020).

### Procedural justice theory

More widely, a substantial body of criminal justice research focuses on procedural justice theory (also referred to as “procedural justice models,” “process‐based models” [e.g., Reisig et al., 2007], and “legitimacy models” [Bennett et al., 2018]), which aims to explain processes that shape citizen's perceptions of authority. Procedural justice theory examines how fair process and the perceived legitimacy of rules and enforcers influence compliance and cooperation with authorities (Moreto & Gau, 2017; Stern, 2008; Sunshine & Tyler, 2003; Tyler, 2006). Proponents of procedural justice theory argue that a central way in which authorities build legitimacy is through the nature of their one‐to‐one interactions with citizens. For example, authorities can enhance peoples’ belief in their legitimacy through legal, consistent, fair, and even‐handed enforcement of laws. In turn, this can generate voluntary compliance, whereby citizens want to comply with and help authorities because they believe that the laws, and those who enforce them, represent their own personal values (Moreto & Gau, 2017; Stern, 2008; Tyler, 2006). A substantial body of empirical research has tested procedural justice theory in the context of police–citizen interactions and found support for many proposed hypotheses. For example, perceived legitimacy of legal authorities has predicted outcomes, including citizens’ willingness to cooperate with police, comply with laws (Sunshine & Tyler, 2003), and accept authorities’ decisions (Tyler & Huo, 2002). Across these studies, there is general agreement that the legitimacy of authorities is influenced by whether citizens believe enforcers act in a procedurally fair manner. Critically, when authorities are perceived as legitimate, citizens voluntarily follow the law because they believe it is the right thing to do (Arias, 2015; Tyler, 2017). This in turn can contribute to more efficient and cost‐effective policing, as incentives or sanctions are not required to secure compliance with every rule or policy enacted (Tyler & Jackson, 2013).

**FIGURE 2:**
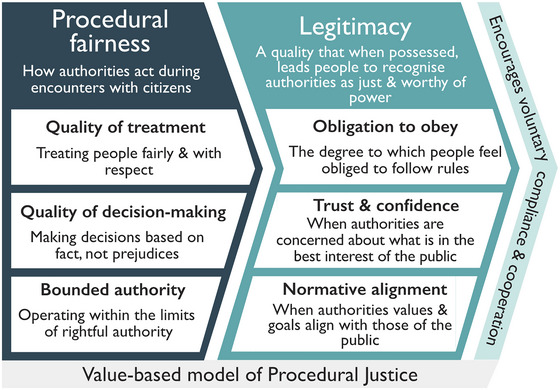
Operationalization of procedural justice theory through the value‐based model of procedural justice in which procedural fairness of enforcers determines their legitimacy and people's law‐related behaviors.

Although some studies have empirically tested elements of procedural justice theory in relation to natural resource management more broadly (Maxwell & Maxwell, 2022; Riley et al., 2018; Schroeder & Fulton, 2017), we are unaware of empirical studies in which interactions between enforcers and citizens were explicitly assessed in protected area contexts with the theoretical model of procedural justice theory as it is applied in criminal justice research (e.g., Trinkner et al., 2018). Yet, we believe doing so could improve the rigor of conservation science and help explain compliance. We drew on a substantial body of criminal justice research to summarize key components of procedural justice theory that have been used to explain compliance and cooperation with police and the courts (Figure 2). In doing so, we aimed to provide guidance to conservationists to support future research on compliance.

## OPERATIONALIZATION OF PROCEDURAL JUSTICE THEORY IN CRIMINAL JUSTICE RESEARCH

Although critics have highlighted inconsistencies in how procedural justice theory has been operationalized in policing studies, prompting the development of validated scales and revised models (Reisig et al., 2007; Reynolds et al., 2018; Tankebe et al., 2016), there is broad consensus that models testing the theory should measure citizens’ opinions of authorities via 2 overarching concepts: procedural fairness and legitimacy.

### Procedural fairness

People are more likely to form positive impressions of authorities and the decisions they make, when they feel that authorities deal with them fairly and respectfully (Huo & Tyler, 2000). Known as procedural fairness, it is typically assessed using 2 core constructs: the quality of treatment and the quality of decision‐making (Figure 2). Quality of treatment evaluates how respectfully, fairly, and courteously people perceive enforcers treat them and the degree to which they believe enforcers respect their rights and listen to their views. Quality of decision‐making captures the degree to which citizens perceive enforcers make decisions fairly, based on fact, and explain their decisions. These, and other constructs, are measured by asking people to report their perspectives using a series of attitudinal statements (Table 1). Procedurally fair encounters with legal authorities have influenced behavior across contexts and actions. For example, procedural fairness was the strongest predictor of obligation to obey the police among a national probability sample of adults in England and Wales (Huq et al., 2017), whereas results from 2 ethnically diverse cities in California indicated procedural fairness was more important in shaping satisfaction with encounters with police and the courts than outcome favorability (Huo & Tyler, 2000). Further, Paternoster et al. (1997) detected evidence of a long‐term compliance effect resulting from perceptions of procedural fairness; men arrested in the United States for domestic abuse were less likely to reoffend when they perceived they were treated fairly.

**TABLE 1:** Overarching concepts and constructs operationalized in studies using value‐based models of procedural justice together with illustrative statements used to collect data; answers are typically reported on 5‐point Likert scales.

Overarching concept and construct	Illustrative question or statement
Procedural fairness^a^	
Quality of treatment	How often do the police treat people with dignity and respect (Trinkner et al., 2018)? How often do the police try to do what is best for the people they are dealing with (Trinkner et al. 2018)?
Quality of decision‐making	How often do the police make fair and impartial decisions in the cases they deal with (Trinkner et al. 2018)? How often do the police make decisions based upon the law and not their personal opinions or biases (Trinkner et al. 2018)?
Bounded authority^b^	When the police deal with people, they almost always behave according to the law (Trinkner et al. 2018). How often do the police respect people's rights (Trinkner et al. 2018)?
Legitimacy	
Obligation to obey	All laws should be strictly obeyed (Tyler & Jackson, 2014). There are times when it is ok to ignore the law (Tyler & Jackson 2014).
Trust and confidence	You generally support how the police act in your community (Tyler & Jackson 2014). The police take bribes (Tyler & Jackson 2014).
Normative alignment^b^	The police generally have the same sense of right and wrong as I do (Jackson et al., 2015). I generally support how the police usually act (Jackson et al. 2015).

^a^ Many refer to this concept as *procedural justice*. However, for clarity, and in line with others (e.g., Jackson et al., 2014), we use the term *procedural fairness* to allow clear distinction between measures of fair process and the overarching theory of procedural justice, in which procedural fairness reinforces legitimacy, which in turn motivates people's law‐related behavior and attitudes.

^b^ Bounded authority and normative alignment are recent additions to theory.

Recent empirical work on procedural fairness has expanded on previous conceptualizations by suggesting the addition of bounded authority, which represents the degree to which citizens perceive enforcers act within or overstep the limits of acceptable authority (Murphy, 2021; Trinkner et al., 2018). Evaluations of bounded authority are not simply determined by the degree to which enforcers follow the law, but rather the degree to which they follow people's normative ideas about the limits to enforcers’ authority. Whether or not people know the legal limits to enforcer behavior is a moot point: different people and different communities vary in their definitions of acceptable enforcer behavior, depending on factors such as culture and history (Trinkner et al., 2018). Boundary concerns significantly influence U.S. citizens’ judgments of appropriate police behavior. When citizens believe police in their community behave appropriately, make fair decisions, and respect the limits of their power (i.e., bounded authority), they are more likely to think they should be obeyed (Trinkner et al., 2018). Similarly, in England and Wales, when members of the public believe police respect the limits of their power, they perceive police as legitimate (Huq et al., 2017). Conversely, overstepping limits to acceptable authority can be detrimental to social order. Muslims in Australia reported greater defiance of police as concerns about officers overstepping the bounds of their authority increased (Murphy, 2021).

We are unaware of studies in conservation or natural resource management that have measured the effect of procedural fairness or boundary concerns related to encounters between enforcers and citizens in line with constructs operationalized in the value‐based models of procedural justice (e.g., Trinkner et al., 2018). However, components of procedural justice theory have been used to examine the trustworthiness of conservation authorities (rather than interactions between enforcers and citizens per se). For example, Maxwell and Maxwell (2022) found that the Banahaw protected landscape management authority in the Philippines was perceived to be more trustworthy, when citizens believe it was impartial and considered their opinions (i.e., procedural fairness). Additionally, where citizens perceived the authority was impartial, they were more willing to comply with regulations (Maxwell & Maxwell, 2022). Procedural fairness similarly predicted hunters’ trust in Michigan State's Wildlife Division (Riley et al., 2018) and anglers trust in, and acceptance of, fisheries management decisions in Minnesota (Schroeder & Fulton, 2017). Conversely, unhappiness with rule makers and perceived illegitimacy of rules stemming from distrust of state authority have been associated with involvement in South Africa's illegal wildlife economy (Hübschle, 2017). Further, recognizing the critical role of institutional legitimacy for effective governance of socioecological systems, Turner et al. (2016) found that trust in information from the Great Barrier Reef Management Authority, assessments of the governing body's performance, and perceived fairness of access to the reef are associated with institutional legitimacy operationalized as support for rules. Indeed, trust is increasingly recognized as integral to effective natural resource management, including when identifying stakeholders’ preferred sources of information (MacKeracher et al., 2018).

Related to bounded authority, several studies have assessed abuse of power by authorities. For example, Sundstrom (2016) assessed the effect of corrupt behavior by officials on the rule‐violating intentions of resource users in a small‐scale fishery in South Africa, finding that resource users were more likely to state rule‐violating intentions when corruption among inspectors was widespread. Meanwhile, when investigating different sanctioning experiences around protected areas in Indonesia and Tanzania, Ibbett et al. (2024) found that the distribution of lawful sanctions by enforcers was perceived to be fairer than interactions that involved abuse of power, such as bribery. Additionally, there is some suggestion that the compassionate use of corruption, for example, turning a blind eye to an offence due to social ties to, or a sense of pity for the perpetrator, can affect how law enforcement authorities are perceived by citizens (Belecky, Moreto, et al., 2021). Further, researchers have assessed the use of discretion by conservation law enforcers when deciding whether to arrest offenders (Eliason, 2003). For example, rangers in South Africa report taking younger perpetrators home to be punished by elders, whereas older offenders are taken to the police (Warchol & Kapla, 2012). Game wardens in the state of Kentucky (USA) similarly applied discretion according to offender age and characteristics including education level and the nature of the violation (Eliason, 2003).

### Legitimacy

From a social psychological perspective, legitimacy is a motivation to act based on positive beliefs about an authority's right to power and influence (Tyler & Jackson, 2013). Legitimacy exists when people believe enforcers have earned the right to command, triggering in themselves an obligation to obey (Hough et al., 2010). Although there has been debate concerning the nature of legitimacy (Reisig et al., 2007; Tankebe et al., 2016), in early studies, it was typically operationalized as a single index containing 2 subscales, one measuring an individual's perceived obligation to obey the law and another quantifying their trust in the relevant authority (e.g., police or the courts) (Table 1; Figure 2). Numerous studies support Tyler's (1990) hypothesis that legitimacy conceptualized in this manner influences peoples’ compliance and cooperation with the police. For example, a study of New Yorkers showed that perceptions of police legitimacy have a stronger influence on citizens’ behavior compared with the risk of being caught and sanctioned (Sunshine & Tyler, 2003). Additionally, data from a national survey of U.S. citizens support the notion that legitimacy has an influence on people's law‐related behavior, whereas risk of sanction does not (Tyler & Jackson, 2014).

Although there is limited comparable work on legitimacy in conservation (by which we mean the application of legitimacy as defined and operationalized in empirical studies of procedural justice theory), there is evidence that psychological factors including descriptive and injunctive norms (St. John et al., 2015; Thomas et al., 2016) and trust in conservation authorities (Stern, 2008) can be influential in shaping compliance. For example, compliance among recreational fishers in the Marlborough Sands, New Zealand, is best explained by intrinsic motivations (e.g., social norms, guilt). These explain 5 times the variance of the model assessing the influence of inclusion in decision‐making on compliance and 4 times the variance of an instrumental model assessing respondents’ perceived probability of being caught and sanctioned (Thomas et al., 2016). Although this study highlights the considerable influence of normative factors on compliance, it suggests inclusion in decision‐making, a process thought to increase legitimacy of rules and motivate compliance, by itself had limited influence on compliance behavior, a finding echoed by McDonald et al. (2020). Data from fishers in Chile similarly suggest that compliance is most strongly influenced by normative, followed by instrumental, factors, and that perceptions of management authority competency and the fairness and appropriateness of rules are not related to compliance (Oyanedel et al., 2020a).

An important extension in the conceptualization of legitimacy is the addition of moral, or normative alignment (Figure 2), which seeks to measure the degree to which respondents perceive that those enforcing rules share the goals, purposes, and values of the community (Jackson et al., 2015; Jackson, Bradford, Hough, et al., 2012; Tyler & Jackson, 2014). Normative alignment embodies a sense of justifiability of power and authority in the eyes of citizens; where views align, people believe their values and the goals of the authorities are similar, thus validating authorities’ possession of power among the public (Hough et al., 2013). Conceptualizing legitimacy as being composed of obligation to obey, trust, and normative alignment (Figure 2), Tyler and Jackson (2014) found that among U.S. citizens, obligation to obey and trust are important predictors of compliance and that normative alignment predicts cooperation with police and courts and people's community engagement. Empirical evidence that normative alignment predicts willingness to cooperate with the police has also been reported among the U.K. public (Jackson, Bradford, Stanko, et al., 2012), whereas research from Sao Paulo, Brazil, established a firm link between procedural fairness and normative alignment with police (Jackson et al., 2022).

In a conservation context, Bergseth and Roscher (2018) measured the strength of normative factors (social norms concerning noncompliance, obligation to obey, moral alignment with and trust in the management authority, and perceptions of fair treatment and decision‐making by the authority) to develop an understanding of the culture of compliance among recreational fishers in Australia's Great Barrier Reef Marine Park. Encouragingly, most fishers perceive the management authority as legitimate and think poaching was socially and personally unacceptable. However, the authors noted, interactions between offenders had the potential, through social learning, to create a poaching subculture. In line with responsive regulation (Ayres & Braithwaite, 1992), they recommend harsher sanctions for repeat offenders (Bergseth & Roscher, 2018).

### Applications of procedural justice theory in non‐Western policing contexts

Many insights from the behavioral sciences, including those on procedural fairness and legitimacy, have been developed in Western, educated, industrialized, rich, democratic (WEIRD) contexts (Crosman et al., 2022; Henrich et al., 2010). Given the global distribution of biodiversity, the relevance of these insights in other contexts is of particular importance (Dorward et al., 2024). The few non‐WEIRD applications of procedural justice theory that exist support the link between procedural fairness and citizen's obligation to obey the police across a wide range of African countries (Levi et al., 2009), whereas perceived legitimacy has also been shown to predict peoples’ willingness to cooperate with police in China (Sun et al., 2017).

However, evidence from South Africa (Bradford et al., 2014), China (Sun et al., 2017), and Pakistan (Jackson et al., 2014) suggests that instrumental variables including perceptions of police effectiveness in fighting crime (how promptly police respond to reported crime and how successful they are at preventing crime, including apprehending offenders [Jackson et al., 2015; Sunshine & Tyler, 2003; Tankebe, 2009; Tankebe et al., 2016]) remain influential. In South Africa, procedural fairness (how enforcers act during encounters with citizens) is important, but perceptions of police effectiveness are more strongly related to people's obligation to obey, perhaps because postapartheid attempts to legitimize the police service were derailed by rapid increases in crime, large uptake in private security, continued experiences of police abuse of power, and low trust and confidence in policing practices (Bradford et al., 2014). In Pakistan, trust in the police is more strongly related to views of police effectiveness than procedural fairness. However, in line with procedural justice theory, procedural fairness still predicts obligation to obey (Jackson et al., 2014). More broadly, there are clear links between respondents’ appraisals of the South African government and police legitimacy; where government performance is in doubt, police legitimacy suffers in this postcolonial, postauthoritarian, postapartheid context (Bradford et al., 2014). Such findings highlight that the wider political, historic, and societal context can affect the legitimacy of conservation law enforcement and that in addition to embedding principles of procedural fairness into conservation law enforcement, conservation policy and practice should be pushing for socially just, ethical, and effective governance of natural resources at all levels.

## IMPLEMENTING THE PRINCIPLES OF PROCEDURAL JUSTICE THEORY THROUGH PROCEDURALLY FAIR ENCOUNTERS

**FIGURE 3:**
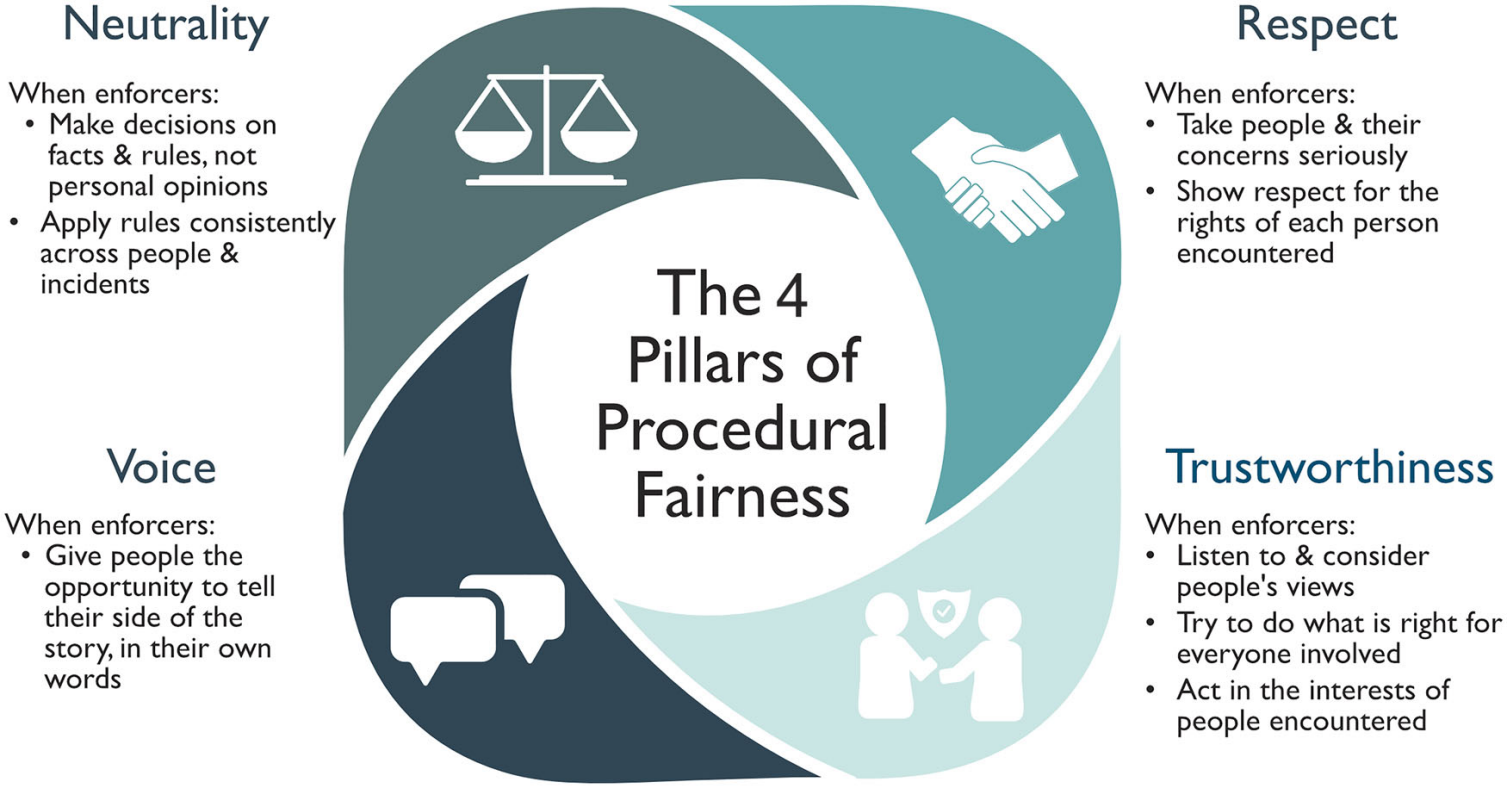
The 4 pillars of procedurally fair encounters with legal authorities: respect, voice, neutrality, and trustworthiness. Adapted from The Justice Collaboratory (2022).

The principles of procedural justice theory can be implemented each time enforcers encounter citizens by embracing the 4 pillars of a procedurally fair encounter: neutrality, which is about fairness in decision‐making; voice, which involves giving individuals the opportunity to participate in the process by telling their side of the story; respect, which is about dignity and involves treating individuals politely and courteously; and trustworthiness, which involves displaying honorable intentions demonstrated by attributes like honesty and transparency in decision‐making (Cohen & Headley, 2023; Mazerolle et al., 2013) (Figure 3). Enforcers who take people's concerns seriously and treat them with dignity and respect, give them the opportunity to tell their side of the story, make unbiased, consistent decisions based on transparent reasoning, and convey trustworthy motives toward those affected by their decisions are more likely to garner legitimacy in the eyes of people, who in turn feel obliged to obey (see Supporting information Appendix S1 for a stylized encounter between an enforcer and an individual found inside a protected area committing small‐scale transgressions). Indeed, procedurally fair encounters may foster positive community relations that can discourage noncompliance (Pendleton, 1998; Sharkey et al., 2024). Training and professional development also provide opportunities to promote core values and interpersonal skills among enforcers. These include acting with integrity (being honest, truthful, accountable); being role models for their profession by respecting the environment, individual rights, cultures, and customs; and leading by example in enabling effective dialogue to create a professional organizational culture (Wildlife Conservation Society, 2023).

Compelling evidence exists that legitimacy and cooperation can be improved through short (<100 s) encounters between enforcers and citizens incorporating the 4 pillars of procedural fairness—respect, voice, neutrality, and trustworthiness (Mazerolle et al., 2013). The potential social benefits of enforcers following pillars of procedural fairness have led to their inclusion in police training in Chicago (USA) (Skogan et al., 2015), Australia (Mazerolle et al., 2013), England and Wales (through professional practice in stop and search [College of Policing, 2017]), and select police units in other countries. Evidence suggests that enforcers wielding authority in a fair, just, and neutral manner within the bounds of their authority hold multiple advantages. These include increased safety and lowered stress levels of enforcers; greater cooperation and diminished defiance from citizens; reduced crime and reoffending rates; and increased voluntary compliance (Sunshine & Tyler, 2003). In turn, such an approach can help tackle more severe crime by encouraging people to cooperate with enforcers and share information with them. This approach can also lead to a withdrawal of support for organized criminal groups.

To date, police forces in various countries have implemented the principles of procedural fairness through community engagement policies, officer and staff training, use of procedurally just scripts during citizen encounters, and application of the pillars of procedural fairness within their organizations (e.g., MacQueen & Bradford, 2017; Wheller et al., 2013; Worden & McLean, 2018). Positively, studies in which researchers used survey or experimental data to assess the effectiveness of training police in the principles of procedurally fair encounters show decreased use of police force and fewer complaints from citizens (e.g., Cohen & Headley, 2023; Wheller et al., 2013; Wood et al., 2020). However, research across several jurisdictions has also identified key roadblocks to implementing such reforms. These include resistance of procedural justice principles among police officers and insufficient organizational coherence. For example, the introduction of procedurally just scripts via top‐down command approaches undermines the theory and does not promote or adhere to internal organizational procedural justice or internal legitimacy (Donner & Olson, 2020; Haas et al., 2015; MacQueen & Bradford, 2017). Other factors found to affect the effectiveness of training include police officers’ interpersonal skills (Fildes et al., 2019) and inconsistencies in the content of training modules and their delivery (Cohen & Headley, 2023).

## CHALLENGES OF EMBEDDING PRINCIPLES OF PROCEDURAL FAIRNESS INTO CONSERVATION LAW ENFORCEMENT

Successfully embedding procedural fairness into conservation law enforcement practice requires enforcers to consistently implement principles during one‐to‐one interactions with citizens. Yet, a range of factors can affect an individual's ability to act in a procedurally fair manner. By its very nature, conservation law enforcement is a dangerous occupation; enforcers frequently risk encounters with dangerous wildlife and potentially hostile, armed offenders (Massé 2019b). Numerous studies highlight that enforcers experience social pressures and repercussions from community members, including threats, abuse, and social ostracism (Anagnostou et al., 2020; Massé, 2019a; Rizzolo et al., 2021). Combined, these factors can contribute to enforcers experiencing stress and anxiety during their duties. Applying models from social psychology, Soofi et al. (2024) studied the nature of interactions between enforcers and citizens around protected areas in Iran and found that frequent negative encounters with illegal hunters (e.g., that involved physical harm or verbal abuse) predict negative affective attitudes toward perpetrators, which in turn are more likely to predict conflict. Studying interactions between game wardens and offenders in Virginia (USA), Carter (2006) found that the demeanor of an offender affects arrest rates, with disrespectful offenders more likely to be arrested. Underlying beliefs, interpersonal skills, and personal circumstances may affect an enforcers’ ability to administer rules consistently and fairly. For example, research from Viet Nam shows that power differentials led national park rangers to adopt attitudes of superiority over the public (Rizzolo et al., 2021). Although there is an emerging body of scholarship focusing on conservation law enforcement, there is a deficiency of research detailing the effect of situationally defined, microlevel factors on enforcer behavior.

At the macrolevel, a significant challenge to effectively integrating principals of procedural fairness into conservation law enforcement is corruption. Defined as the use of public office for private gain (Wilson & Damania, 2005), some of the most biodiverse places on earth suffer some of the highest levels of corruption (Transparency International, 2022). Corruption undermines citizens’ perceptions of government honesty and competence and affects willingness to comply with laws (Levi et al., 2009). Evidence from 18 African countries and Pakistan shows that individuals’ perceptions of authorities’ effectiveness, trustworthiness, and legitimacy are reduced when authorities engage in illegal acts, such as bribery (Bradford et al., 2014; Jackson et al., 2014; Levi et al., 2009). The negative implications of corruption on conservation have long been noted (Robertson & Van Schaik, 2001; Smith, Obidzinski, et al., 2003), with recent localized survey data (Kisingo et al., 2022; Sundström, 2012) confirming trends identified at wider spatial resolutions (e.g., pan‐African) (Hauenstein et al., 2019; Smith, Muir, et al., 2003). Systemic organizational problems, such as low pay, few motivational incentives (including timely promotion and sufficient rewards), and minimal supervision, affect conservation enforcer misconduct, which in turn can undermine citizens’ perceptions of enforcers’ legitimacy (Belecky, Moreto, et al., 2021; Kisingo et al., 2022; Ogunjinmi et al., 2008). Addressing such factors with limited funds is challenging, meaning conservation agencies may struggle to simultaneously tackle staff corruption and maintain the necessary workforce for effective enforcement.

This conundrum further highlights the pressing need to reconceptualize conservation law enforcement away from enforcer‐coerced compliance and toward strengthening a wider base of voluntary compliance (Arias, 2015). Incentives for corruption may be reduced by ensuring basic needs of enforcers are met through sufficient and prompt pay, and adequate provisioning of equipment (Belecky, Parry Jones, et al., 2021). However, when dealing with powerful offenders, fair administration of the law can be difficult to enact (Ibbett et al., 2024). Financial, physical, or psychological threats can make it safer for enforcers to collude (Miller, 2011), and where corruption is institutionalized, or positions of power are maintained through patronage, pressure from superiors can make it impossible to avoid (Belecky, Parry Jones, et al., 2021; Paley, 2015). Consequently, development of anticorruption policies requires an understanding of relationships between actors, alongside reform of political systems and enforcement infrastructure (Wilson & Damania, 2005).

Further, the legitimacy of authorities is influenced by political issues including colonial legacy, community tensions, marginalization, and state abuse of power. These factors can erode trust in state authority, making it harder for policies and interventions, including laws and their enforcement, to be effective (Beetham, 2013). Moreover, the impacts of displacement and colonization associated with the way conservation has been implemented in many places hold long‐lasting implications for relations between citizens, conservation authorities, practitioners, and scientists (Dore et al., 2022). In such contexts, wider structural changes, beyond improving encounters between enforcers and citizens, are required, especially because compliance is more readily achieved where laws align with people's sense of what is right or wrong (Murphy et al., 2009). Moving forward, conservation policy and practice must acknowledge past structural, systemic, and individual injustices inflicted. To this end, much may be learned from conservation projects incorporating principles of environmental restorative justice (Hübschle et al., 2021).

## ENHANCING CONSERVATION LAW ENFORCEMENT THROUGH PROCEDURAL JUSTICE

Reconciling these realities requires a step change in conservation law enforcement. If people view rules and their enforcers as unfair or corrupt, they will not recognize them as legitimate; the result is mistrust and noncompliance (Moreto & Gau, 2017). Although history can affect an authority's claim to legitimacy (Dore et al., 2022; Tankebe, 2009), procedurally fair actions of enforcers can improve citizen's relationships with legal authorities. Enforcers performing their duties in a procedurally fair manner becomes increasingly important in shaping compliant behavior in conflict scenarios where people question the legitimacy of laws (Murphy et al., 2009). Procedural considerations including participation, recognition, and justice contribute to robust environmental governance (Bennett & Satterfield, 2018) likely because inclusion in governance satisfies human's sense of agency (DeCaro & Stokes, 2013). Indeed, a degree of autonomy in making and enforcing rules have long been identified as an important feature limiting exploitation and sustaining resources (Ostrom et al., 1999). This makes procedural justice theory particularly relevant to conservation policy and practice, both in rule formulation (Ruano‐Chamorro et al., 2022) and implementation.

Incorporating principles of procedural fairness into interactions between enforcers and citizens represents a more democratic, less harmful way of policing that, in the long term, has the potential to increase voluntary compliance (Trinkner et al., 2018). Although achieving sufficient levels of voluntary compliance will be challenging, particularly in regimes where there has been little previous experience of respect for human rights, democracy, and the rule of law (McEvoy et al., 2017), ultimately increased self‐regulation has potential to reduce the enforcement focus of patrols. This releases resources for other activities, be they training, improved wages and working conditions, strengthening community relations, or other activities essential to the ecological integrity of protected areas. In achieving this, much can be learned from the field of environmental justice, which offers a theoretical foundation to investigate justice in environmental change and management and similarly considers other concepts, such as procedural justice, morality, and distributional and recognitional justice (i.e., how costs and benefits are distributed between groups, and whose views and interests are legitimized) (Lau et al., 2021).

There is evidence from conservation that value‐based motives including norms and trust can be more effective at securing compliance with conservation rules than strategies based solely on deterrence (Oyanedel et al., 2020b; St. John et al., 2015; Stern, 2008). Although current conservation policy has repeatedly called for more boots on the ground (Appleton et al., 2022), we argue this alone will be insufficient to generate greater compliance with conservation laws. Conservation practice must address challenges associated with the fairness of laws, as well as fairness in their administration (Ibbett et al., 2024). To this end, ratification and adoption of the International Ranger Federation's (2021) Ranger Code of Conduct and wide recognition of the need to professionalize conservation law enforcement by tackling problems, such as corruption, and promoting healthy relationships between enforcers and local people are positive steps forward. Additional steps may include learning from general policing and diversifying enforcer training beyond traditional topics of patrolling, intelligence gathering, crime scene processing, surveillance, paramilitary training, and law and legal processes (Warchol & Kapla, 2012). Any such training will need to be integrated into authorities’ standardized operating procedures and should provide opportunities for umbrella organizations, such as the International Union for Conservation of Nature and the International Ranger Federation, to continue supporting the development of minimum standards and codes of conduct. Current efforts that support enforcers’ learning on human rights, and the makeup of procedurally fair encounters, provide opportunities to reflect on core principles from a philosophical (e.g., how should humans treat each other) and legal perspective (Wildlife Conservation Society, 2023). Greater integration of social safeguard approaches that increase the accountability of law enforcement personnel and protected area authorities, such as formal complaints procedures, grievance redress mechanisms, and specific reviews after use of force (Wilkie et al., 2022), would further complement efforts to establish a culture of law enforcement grounded in procedural justice theory. All efforts would benefit from robust impact evaluation to strengthen the evidence base and ensure that conservation is investing in effective strategies.

## CONCLUSION

Currently, conservation is reliant on huge investments in law enforcement to secure compliance, particularly around protected areas. Yet, people's decisions to commit infractions are often complex and context dependent (Bergseth & Roscher, 2018). In many protected area contexts, conservation law enforcement has invested in increasing the cost of noncompliance and reducing opportunities for offending to occur. Yet, other models of enforcement highlight that procedural fairness and the legitimacy of enforcers and the laws themselves are also important factors that can influence compliance. Conservation law enforcement has been studied in numerous ways, including through examinations of how sanctions affect behavior (Milner‐Gulland & Leader‐Williams, 1992; Wilson & Boratto, 2020) and how resource allocation motivates enforcers (Jachmann, 2008). Yet, how rules are enforced is also important because it affects how people respond to specific conservation rules and conservation more generally. In general policing, research operationalizing procedural justice theory through the value‐based model of procedural justice provides substantial evidence that when enforcers treat citizens fairly and respectfully as human beings, give citizens a voice, and are objective and respectful, they gain peoples’ trust and gain legitimacy, both individually and for the institution they represent. In turn, people are more inclined to comply voluntarily with laws, cooperate with enforcers, and help them (Sunshine & Tyler, 2003), thereby reducing dependence on coercive control. Research grounded in this value‐based model of compliance can highlight how current approaches to conservation law enforcement could be complemented and improved. Alongside ensuring the integrity and accountability of conservation law enforcement (International Ranger Federation., 2021), embedding principles of procedural fairness into encounters between enforcers and citizens has the potential to reduce low‐level noncompliance, particularly in protected area contexts. Success, however, requires a reconceptualization of conservation law enforcement that places instrumental and value‐based drivers of compliance on a more equal footing both in research and practice.

## Supporting information



Supporting Information
