# Supplementary material for: Using lessons from criminal justice research to improve conservation law enforcement research and practice
Source: Conserv Biol. 2025 Jul 3;40(1):e70094. doi: 10.1111/cobi.70094 (PMC7617914; doi:10.1111/cobi.70094)
Supplement: Supplementary file 1 — Supporting Information [file COBI-40-e70094-s001.pdf]

Using lessons from criminal justice research to improve conservation law enforcement research and practice

St John et al., (2025)

Appendix S1
Consider two stylized encounters between an enforcer and an individual found inside a protected area committing small-scale transgressions for which they receive a fine. In the first, the enforcer (1) exhibits aggressive non-verbal cues via their body language and attire, foregoes customary greetings, and (2) neglects to say why they have stopped the person. (3) They fail to give the individual an opportunity to tell their side of the story, and (4) issue a higher fine than they have issued to others for the same offence, whilst making racial slurs about the assailant's background. Lastly, they do not explain the reasoning underpinning their decision to issue the fine. In the second encounter, the enforcer (1) exhibits calm body language, greets the rule-breaker according to local customs and explains why they have stopped the individual, before (2) asking them why they were inside the protected area. (3) The enforcer issues the same fine awarded to others caught committing the same offence, and (4) explains to the individual the reason for them being fined. With professional demeanour, the enforcer also informs the individual of the consequences of further infractions.

Compounded by the enforcer's aggressive body language and attire, the first encounter depicts negative interpersonal treatment, unfair decision making and disregard for the boundaries of authority. Conversely, the second encounter epitomises the four pillars of procedural fairness: (1) Respect, (2) Voice, (3) Neutrality and (4) Trustworthiness (Figure 1). Throughout the encounter, the enforcer treated the rule-breaker with respect and dignity and by listening to their side of the story, gave them voice. By issuing the same fine given to others committing the same infraction, and explaining their decision, the enforcer conveyed neutrality and transparency to the individual. Moreover, by explaining consequences of further infractions, the enforcer expressed concern about the well-being of those impacted by law enforcement. The actions of the enforcer conveyed value and status to the rule-breaker, which in turn strengthens legitimacy of authority. Whilst both were fined, the individual depicted in the second encounter will be more accepting of the penalty than the person described in the first, because they would assess their penalty as fairly arrived at (Tyler 2017). Knock-on effects include greater compliance, cooperation and empowerment of the individual enforcer and the conservation authority more generally (Jackson et al. 2015).

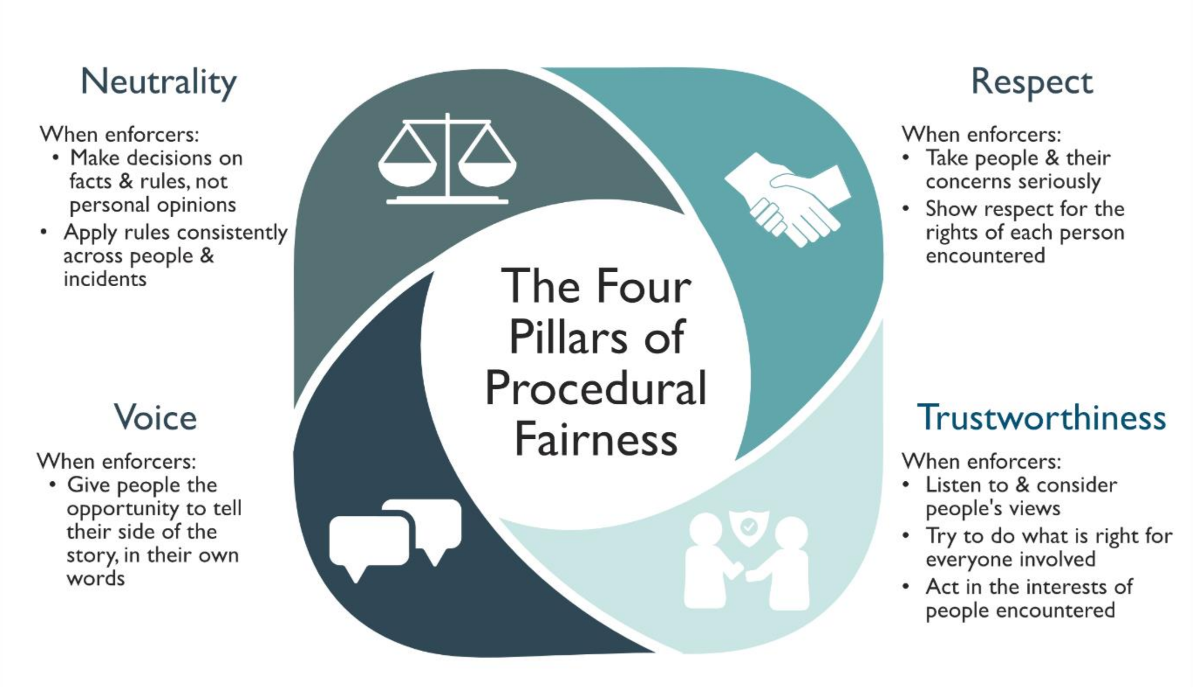

Figure 1. The four pillars of procedurally fair encounters with legal authorities: respect, voice, neutrality, and trustworthiness. Adapted from The Justice Collaboratory (2022).

References

Jackson J, Hough M, Bradford B, Kuha J. 2015. Empirical Legitimacy as Two Connected Psychological States. Pages 137–160 in Meško, G., Tankebe J, editor. Trust and Legitimacy in Criminal Justice. Springer, Cham. Available from https://doi.org/10.1007/978-3-319-09813-5_7.

Tyler TR. 2017. Value-Driven Behavior and the Law. Pages 403–422 The Oxford Handbook of Law and Economics: Volume 1: Methodology and Concepts.
